# Data pipeline for managing field experiments

**DOI:** 10.1016/j.mex.2023.102031

**Published:** 2023-01-19

**Authors:** Jian Liu, Rogerio Cichota, Stephanie Langer, Eric Burgueño, Alexandre Michel

**Affiliations:** The New Zealand Institute for Plant and Food Research Limited, New Zealand

**Keywords:** Real-time visualisation, Experiment management, Data streamlining, Data pipeline for managing field experiments

## Abstract

As agricultural and environmental research projects become more complex, increasingly with multiple outcomes, the demand for technical support with experiment management and data handling has also increased. Interactive visualisation solutions are user-friendly and provide direct information to facilitate decision making with timely data interpretation. Existing off-the-shelf tools can be expensive and require a specialist to conduct the development of visualisation solutions. We used open-source software to develop a customised near real-time interactive dashboard system to support science experiment decision making. Our customisation allowed for:

• Digitalised domain knowledge via open-source solutions to develop decision support systems.

• Automated workflow that only executed the necessary components.

• Modularised solutions for low maintenance cost and upgrades.

## Graphical abstract

**Figure:**
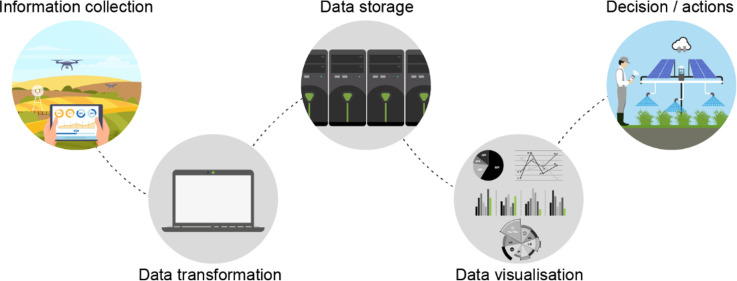

Specifications table

**Table:** 

Subject Area:	Agricultural and Biological Sciences
More specific subject area:	*Field and indoor environmental data collection for agricultural research projects*
Method name:	*Data pipeline for managing field experiments*
Name and reference of original method:	*W.M. Landau, The targets R package: a dynamic Make-like function-oriented pipeline toolkit for reproducibility and high-performance computing, J. Open Source Softw. 6 (57) (2021) 2959, doi:*https://doi.org/10.21105/joss.02959
Resource availability:	*Hardware:* (1) *Neutron probe soil moisture sensor* (2) *GreenSeeker device* (3) *Linux server* *Software:* (1) *Microsoft Excel* (2) *Microsoft SharePoint* (3) *Rstudio* (4) *R* (5) *PostgreSQL* (6) *Grafana* (7) *Docker*

## Introduction

Transforming data into information and then into actions often demands the integration of a large number of tools to organise, clean up, store, analyse, and visualise data [1,2]. The onset of technologies grouped under terms such as Internet of Things (IoT) and ‘big data’ have ushered an increased need for data-driven methodologies to study and manage farming systems [2,3]. The ever-increasing volume and variety of data demands particular computational skills and the use of a wide range of tools [4,5]. This also involves the integration of tools considering the specific needs of different disciplines as well as the requirements for different stages of data processing (e.g. for data collection, analysis, and visualisation). A variety of frameworks to formalise this integration, as well as workflows and pipelines to link the many tools available, have been proposed [1,[6], [7], [8]] and many are in development. More recently, and in the same space, the concept of ‘digital twin’ has been introduced. This involves combining measurements with simulation or machine learning models and data analysis to mimic *in-silico* a given system (a plant, a farm, or even a whole sector). It also comprises real-time interactions with the actual system to develop a more holistic understanding as well as to control it [9,10]. This concept uses technologies such as IoT and requires similar tools and skills to handle large and varied datasets, but goes beyond visualising and understanding data, aiming to actively monitor the system and trigger management actions.

These continuous technological advancements constantly challenge how scientific work is planned, conducted, and communicated [5]. Research projects are becoming more complex over time, accounting for a wider range of interactions, and requiring cross-discipline engagement [6,11,12]. In agricultural and environmental research, projects need to increasingly focus on multiple outcomes (e.g. productivity, quality, resource constraints, and impacts on the environment) and thus require tracking and analysing a wide range of variables. Field experiments and monitoring are commonly used in agricultural and environmental research, but they are often difficult and costly to conduct. Because of the complexity and variability of natural and agricultural environments, it is hard to control the conditions of field trials and to collect representative data in large volumes. The use of IoT technologies offers a promising prospect to increase the amount and breadth of data collected, with higher spatial and temporal resolution and encompassing a wider number of variables [2,6]. Importantly, the ever-increasing complexity of experimentation and the volume of data being collected require support systems that can help researchers monitor trial performance, make decisions on managing trials, and analyse results [2,8].

Here, we present details of a pipeline to process, store, and examine data collected in field experiments at The New Zealand Institute for Plant and Food Research Limited (PFR). We describe the tools and techniques used to develop the pipeline, and demonstrate its application on a field trial helping to manage irrigation and guide data collection. In particular, we deal with handling manually collected data and describe our approach to standardise and streamline analyses. Our objective is to contribute towards an integrated IoT system to support scientific experiments. This includes the collection and manipulation of data to facilitate the management of trials and to aid the subsequent analyses and interpretation of results. We envisage that this methodology could be embedded into a ‘digital twin’ system for research at PFR.

## Method details

### Rationale

Conventionally, trial management and data verification are often made by the domain scientists investigating the data themselves while conducting field experiments. However, with ever-increasing complexity of agricultural and environmental research, the conventional approach faces three main challenges. Firstly, scientists are often under time pressure to produce analyses for guiding the operation team to take actions for trial management. This includes, for instance, turning irrigation on to prevent plants from suffering unduly water deficiency, or collecting leachate samples following a sufficient amount of drainage. Secondly, experiments increasingly involve multiple objectives and multi-disciplinary teams, requiring more sophisticated forms of visualising complex information to ensure all involved have an understanding of experiment status. Thirdly, details and data collection mishaps may be easily overlooked when the volume of data is large and urgent decision making is required. This may result in unamendable mistakes or delayed corrective actions that can lead to loss of information/data.

We have developed a workflow and implemented a customised pipeline for data manipulation and visualisation for use in scientific field experiments within our research institute. The base principles of this workflow were:

(1) Engage with domain scientists and operational team to clarify the science questions and measurements required to answer those questions.
(2) Define techniques and plan the general sampling strategy ahead of the peak data collection period;
(3) Establish and standardise data entry tables and protocols with key stakeholders.
(4) Engage with domain scientists and operational team to design a fit for purpose information workflow.
(5) Use near real-time visualisation as the interface to interact with key stakeholders and collect feedback for continuous improvements.

We found that the implementation of customised data pipelines was accelerated by using these generic principles to guide the requirement gathering process. These generic principles can be adapted for different types of science questions, and therefore, different data.

The current workflow focuses on handling soil water and plant cover data that are collected and entered into the pipeline manually.

### Workflow description

**Fig. 1:**
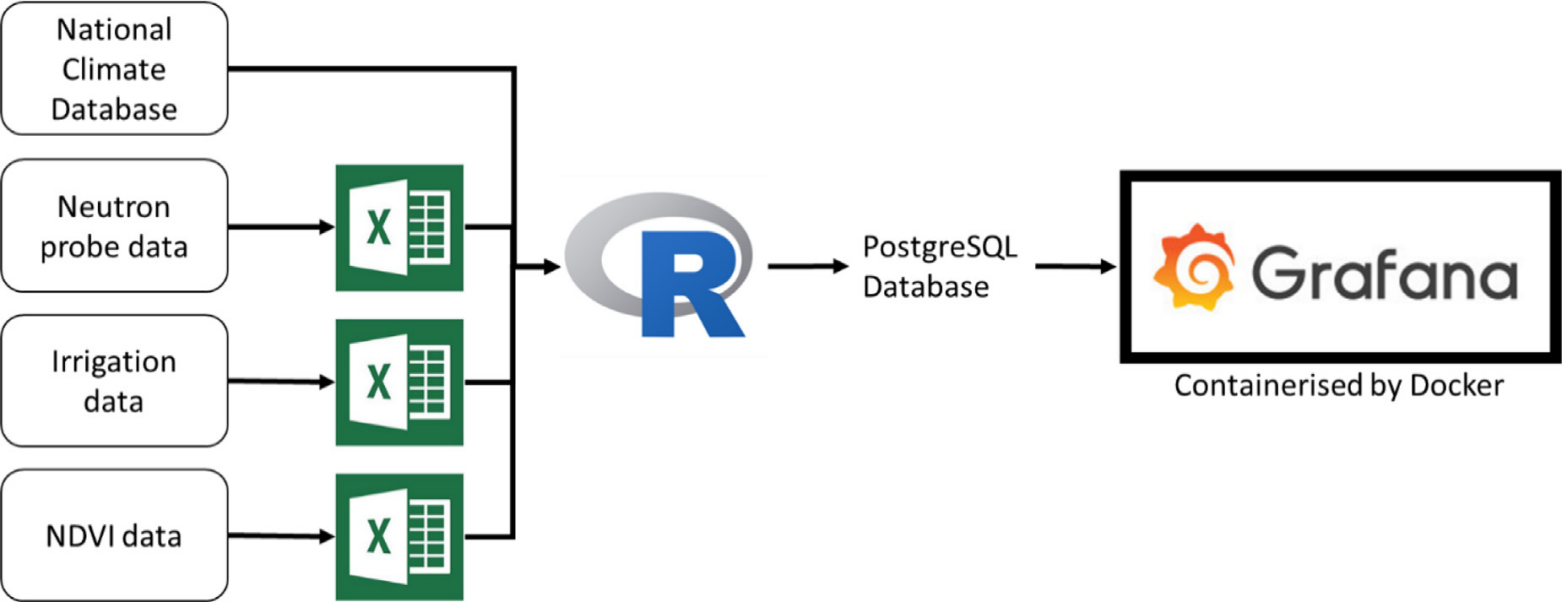
A technology overview of the customised workflow to visualise manually collected data from field experiments in near real-time with weather data obtained via application programming interface (API). Black arrows indicate data flow directions. Weather data from the experimental site's nearest weather station in the NIWA National Climate Database were queried by API. Soil moisture content, irrigation application rate and normalised difference vegetation index (NDVI) data were exported from the instruments and manually uploaded to different Microsoft Excel spreadsheets on the institute level SharePoint server by an operational team member. Data were merged and aggregated in R based on the domain knowledge obtained from experts. Aggregated data were streamed into a PostgreSQL database on the institution server. Grafana, a ready-to-use dashboard software, was used to visualise the data interactively. Docker container was used to host the Grafana application.

A schematic with the general workflow is shown in Fig. 1. This focuses on handling soil water and plant cover data that are collected and entered into the pipeline manually. The workflow encompasses handling information from the essential data sources to the visualisation solution (Grafana [13]). The decision-making phase was deliberately omitted because our aim was to assist the stakeholders to make informed decisions. Nonetheless, expanding the pipeline further to include triggering alerts or management actions is plausible and is currently under development. With the machine-aid mindset, a planning workshop was held to structure the key metrics to visualise and layout the steps for achieving the target. A customised plan of data orchestration and manipulation was developed to achieve the three following tasks:

(1) Data acquisition. The data acquisition script has two purposes. Firstly, it imports the soil water content (SWC), normalised difference vegetation index (NDVI) and irrigation data from Microsoft SharePoint to the R environment, and extracts the date information to instruct the application programming interface (API) to query the relevant weather data. Secondly, the script sends queries to the NIWA National Climate Database [14] and retrieves potential evapotranspiration (PET) and daily rainfall data.
(2) Data transformation. Thirteen R functions were developed to capture and implement the domain knowledge for relating different data sources together. In brief, the functions summarised raw soil moisture data and manual NDVI data to obtain the moisture status over the soil profile and estimate the canopy coverage over the plant growth period. Next, we employed a modified water balance algorithm based on the work of Horne and Scotter (2016) [15] to incorporate irrigation, rainfall, PET and NDVI data to predict soil moisture deficit and potential drainage.
(3) Database update. A delegated PostgreSQL database was setup by the IT department to store the summarised data. An R function was written to upload tabulated data to the databases for subsequent visualisation.

### Pipeline implementation

**Fig. 2:**
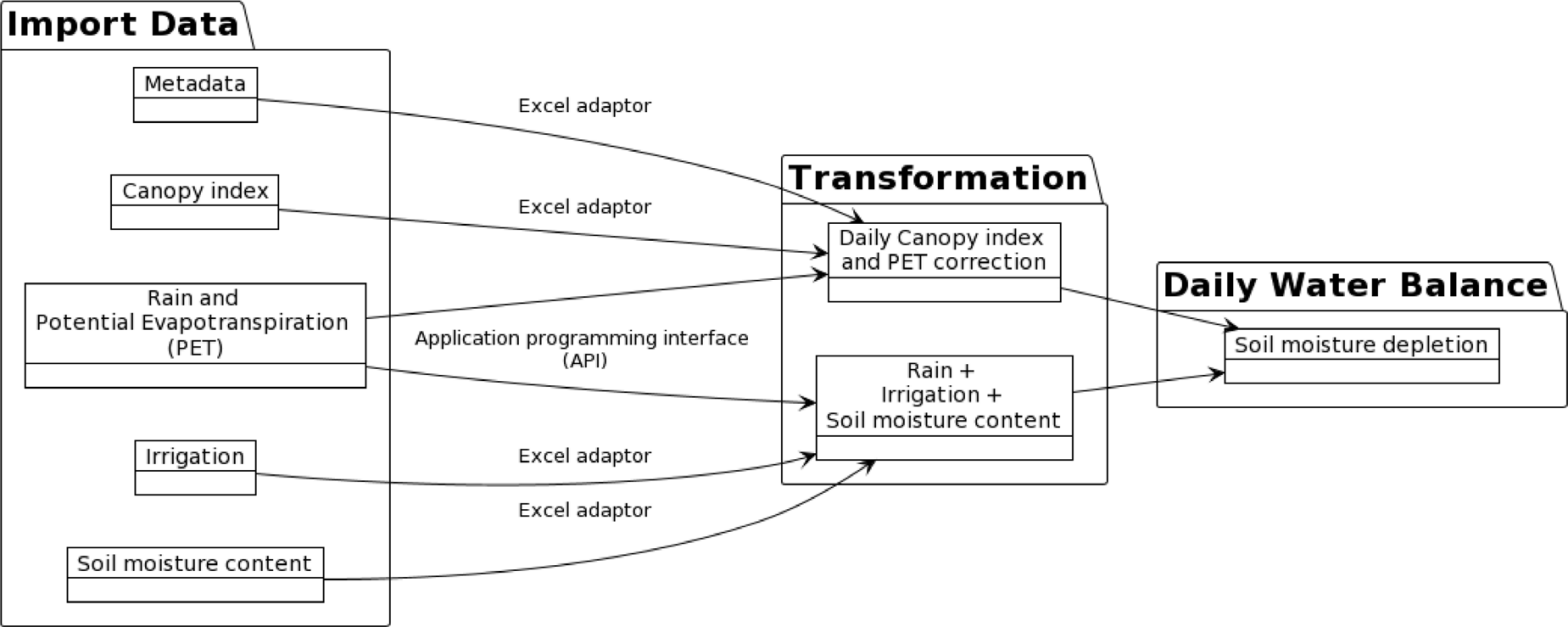
A simplified directed acyclic graph (DAG) that represents the R data pipeline. The detailed R scripts with documentations are hosted on GitHub repository (https://github.com/frank0434/MethodXCodeRepository). More detailed website links in Supplementary Table 1. DAG was created by PlantUML [18].

Fig. 2 illustrates a simplified data pipeline that we implemented in R by using the *targets* package [16] (detailed pipeline diagram in Supplementary Fig. 1). The data pipeline contains 15 functions that were stored in the “functions.R” script with their customisation details. These functions were customised to achieve five main functionalities including data source connection, meteorological data retrieve, canopy data retrieve and processing, water balance calculation, and utility. Detailed explanation as follows:

(1) The data source connection group has two functions (function 1 and function 2). Function 1 is the Excel adaptor (Fig. 2). This function is responsible for accessing Excel files on the shared server that is protected by Windows New Technology LAN Manager (NTLM). Credentials are stored in the R environment file in the user's private directory and sourced automatically by built-in R functions. This strategy was adapted to avoid manually typing credentials each time. The second function facilitates the PostgreSQL database connection and summarised data uploading (Supplementary Fig. 1). The same authentication strategy was used for the second function.
(2) Another function was written to retrieve the meteorological data (function 3). This function wrapped the R package clifro [17] and automated the Rain and PET data based on the experiment duration.
(3) The canopy data group has two functions (function 4 and function 5). Canopy data were stored in multiple Excel files on the Microsoft SharePoint server. Different instruments were used for canopy measurements because of practical issues during the experiment periods. Furthermore, it is essential to take into account the fallow period and crop canopy development stage. This is because the actual evapotranspiration is often less than the PET before full canopy closure. Moreover, canopy measurements were only performed fortnightly, or less frequently in some occasions. Therefore, it was necessary to standardise (function 15) and interpolate (function 8) the data to daily values. We made two critical assumptions for processing canopy measurements. Firstly, the canopy development had a linear relationship with time between emergence and canopy closure. Secondly, the canopy closure was achieved at the NDVI value of 0.75 for all crops. These assumptions were justified by the field observations for the vegetable crops that receive adequate or surplus water.
(4) The water balance calculation group has eight functions (function 6 to 13). The critical function (function 13) contains the implementation of the water balance equations [15]. Function 6 to 12 were designed to prepare the water-related data to meet the input requirements of function 13. We intended to modularise the function to obtain the essential soil moisture metrics. For example, a simple and practical approach to estimate the field capacity across the soil profile is by assuming that the field capacity soil moisture content is reached during fallow period in winter, when the evaporation is minimal. Function 12 was written to achieve this estimation.
(5) Two utility functions, function 14 and 15, were used to modify time variables and perform lag time calculation, respectively.

Another R script was used to trigger the *targets* data pipeline (CodeSnippets 1). This script is made available as an executable programme in the laboratory computers that host the interfaces to the data tables and other scientific instruments. Therefore, the operations team members can execute the programme immediately after they complete the manual data entry, and then evaluate the visualisation of all measurements to decide whether an action is required.

CodeSnippets 1 – An example for using R executable to call an R script that triggers the workflow:

### Deployment and operation

The main R scripts were developed in the RStudio (version 1.4.1717) environment. The R scripts were then executed using the laboratory computer with an environment file that contained credentials for file and database accesses.

One challenge of using R in a production environment is the reproducibility of the scripts across platforms and environments. We used the R-package *renv* to tackle this challenge [19]. The key function of *renv* is to create a specific file called *renv.lock* to record package dependencies and version numbers. Once the environment is created, it is possible to share the lightweight environment file with the associated folder via cloud Git hosts such as GitHub or Bitbucket, or even copy and paste onto flash drivers when internet access is limited.

### Visualisation component

We used Grafana as the visualisation solution. This was mainly a time-saving consideration from user interface (UI) design to ensure the data transformation script was robust and reliable. As an off-the-shelf UI solution, Grafana is designed to visualise large volumes of time-series data with drag and drop visualisation components, and built-in interactive features. Grafana was deployed as a service that is available to all internal users via a uniform resource locator (URL). The development was completed by using the docker-compose command on a Linux server (CodeSnippets 2).

CodeSnippets 2 – The YAML file to instruct the deployment of Grafana as a service via docker-compose command (detailed explanations are in the supplementary material):

CodeSnippets 3 shows the actual command in the Linux server to build the Docker container for hosting Grafana. The *-d* flag was used to configure the container running in the background. We acknowledge that the Docker software in the Linux server may require extra effort from the IT department or individual researcher to install and maintain. Alternatively, free desktop solutions for individual personal use can be also be used. The desktop solution demands the minimum effort for deploying applications. However, a downside is limited access by other developers.

CodeSnippets 3 – The command and flag to deploy Grafana as a website application in Docker container:

Once the container is deployed, it is accessible via the Linux server URL or localhost with the ports defined in the docker-compose file (Ports in CodeSnippets 2). More specifically, the access URLs should be in the following formats:

(1) Internal Linux server: http://<name or IP address>:3000/.
(2) Desktop Docker: http://www.localhost:3000/.

The Grafana application requires a minimum of two steps to display visualisations. Firstly, users must define a data source for the application to retrieve data. Secondly, users have to assemble the desired dashboard widgets by using the drop and drop features. A detailed walk-through can be found in the supplementary material (Supplementary Information 1).

**Fig. 3:**
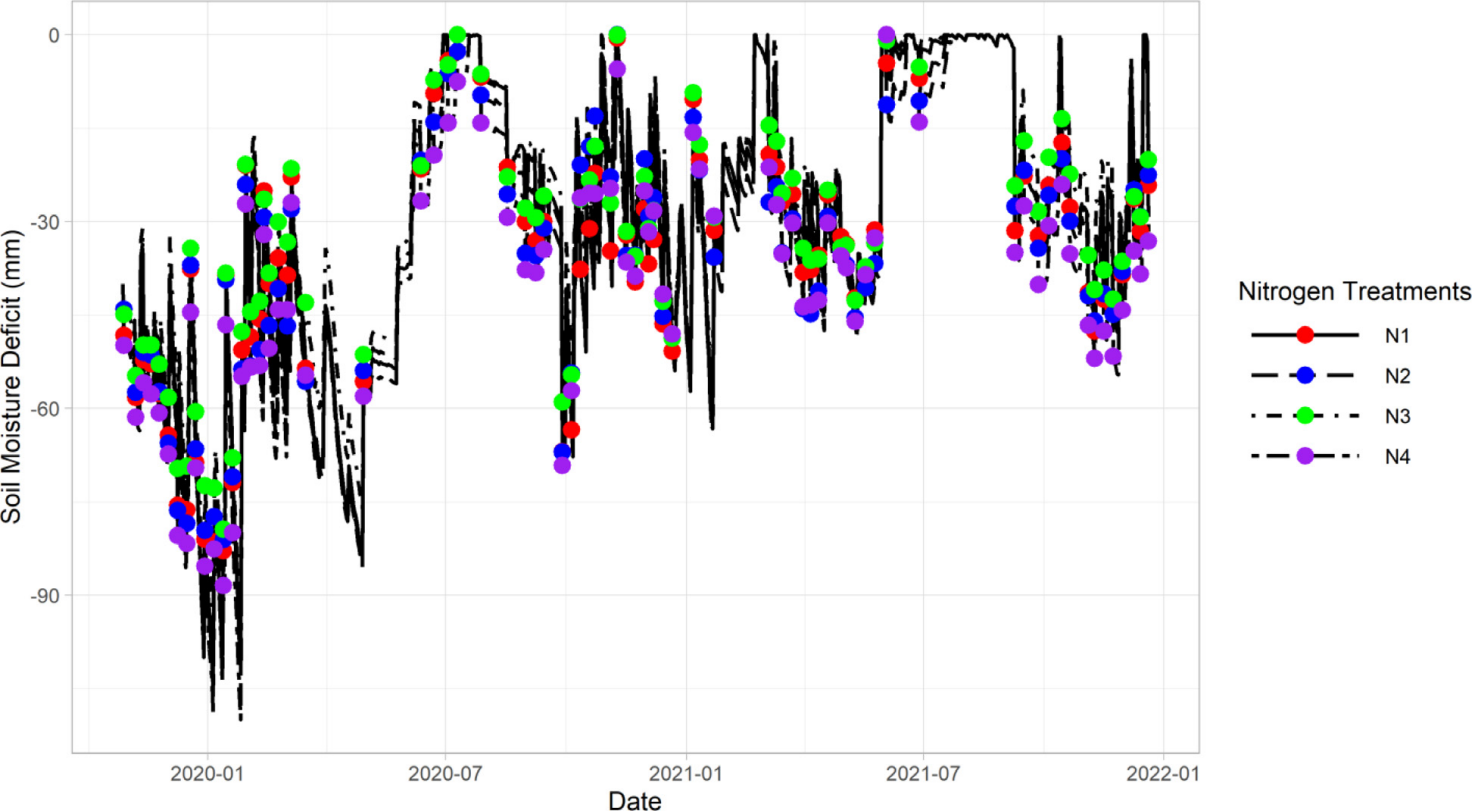
Final visualisation of soil moisture deficit (SMD; mm) in the top 60 cm soil profile to allow the stakeholders to make decisions on irrigation application rates for four nitrogen treatments. Nitrogen treatments, N1 (red points), N2 (blue points), N3 (green points) and N4 (purple points) represent control (no nitrogen applied), half rate of Good Nitrogen Management Practice (GNMP; industry standard), GNMP and double rate of GNMP, respectively. Black lines show the predicted SMD values by the water balance equation. The prediction was corrected by resetting the value to observed values and adjusting PET to canopy cover measurements. The correction was performed whenever new observation values were available. Please note that this figure is a static graph that was created in R for publication purposes. The interactive version in the Grafana user interface (UI) had a slightly different appearance.

Fig. 3 demonstrates an exemplar of the final visualisation for soil moisture deficit (SMD). The SMD was corrected by two factors. Firstly, soil water balance was reset to the observed soil moisture content whenever available. Secondly, PET values were adjusted by the fraction of canopy cover index (range between 0 and 1; 0 indicates no crop canopy cover and a reduced PET value was used, while 1 represents full canopy cover and full PET values were used).

**Fig. 4:**
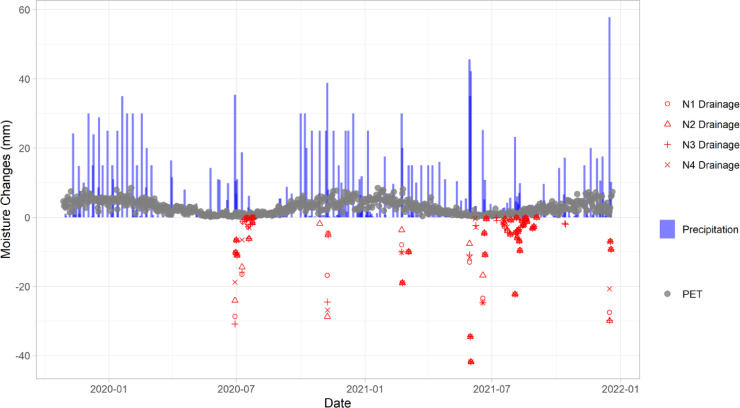
Moisture changes output from soil water balance equation. Nitrogen treatments, N1 (red circle points), N2 (triangle points), N3 (plus points) and N4 (cross point) represent control (no nitrogen applied), half rate of Good Nitrogen Management Practice (GNMP; industry standard), GNMP and double rate of GNMP, respectively. Red colour indicates the prediction of drainage from four nitrogen treatments. Drainage predications were based on the soil water balance model with two corrections: (1) resetting soil water content whenever new observations are available, and (2) adjusting potential evapotranspiration (PET) based on the daily interpolation of normalised difference vegetation index (NDVI) values measured for the different crops. Please note that this figure is a static graph that was created in R for publication purposes. The interactive version in the Grafana user interface (UI) had slightly different appearance.

Calculated drainage events are shown in Fig. 4. Red symbols with the label prefix “N” represented different events in four different nitrogen treatments. In general, drainage events occurred either after the precipitation exceeded 20 mm during the winter period (June to August, when PET is low) or consecutive precipitation (over 20 mm) in the spring. The interactive UI allows users to see the exact values; however, the feature is omitted in the static demonstration. This information was highly valuable for taking decisions regarding when to collect N leaching samples and was also used to help managing the irrigation. For the science team, visualisations like those of Fig. 3 were found useful to verify differences between treatment as well as the variability among replicates in near real-time, which supported discussions on experiment management and potential adjusts to them or future analyses.

## Conclusion and future work

We have implemented a customised targets pipeline consisting of domain knowledge, PostgreSQL database and Grafana to aid monitoring and on decision making in field experiment management and anomaly detection for a science project. The workflow successfully delivered near real-time data integration and visualisation. The workflow has performed reliably and the operations team found it to be useful to facilitate the decision making on irrigation rates, sampling time, and data quality verification. However, we do acknowledge that there is room for improvement. For example, the pipeline has not been implemented into handling large amounts of data (more than gigabytes). Therefore, the feasibility of such pipeline on big data that is generated from automated collection remains unknown. Another possible improvement is to extend the capability to the IoT for automated irrigation events without human intervention. Regarding the science quality, established domain knowledge bases can be incorporated to leverage extensive understanding of the mechanism of the soil solute movements.

## Declaration of Competing Interest

The authors declare that they have no known competing financial interests or personal relationships that could have appeared to influence the work reported in this paper.

## Data Availability

The authors do not have permission to share data.

## Appendix Supplementary materials

Image, application 1
