# Supplementary material for: Data pipeline for managing field experiments
Source: MethodsX. 2023 Jan 19;10:102031. doi: 10.1016/j.mex.2023.102031 (PMC9922957; doi:10.1016/j.mex.2023.102031)

Supplementary Figure 1. The directed acyclic graph (DAG) visualises the R objects that involve in the analytic pipeline. Each dark green node represents an R object. The arrows indicate the information flow. More specifically, multiple arrows pointing to one node indicate that the node depends on multiple inputs. On the contrary, the node is dependent on multiple subsequent nodes when one node has multiple arrows. The graph was generated by the R function ` tar_visnetwork(targets_only = TRUE)` in the *targets* package. Nodes will be displayed in red colour when errors occur. Other status indicators exist. Please refer to the help documentation for more details.


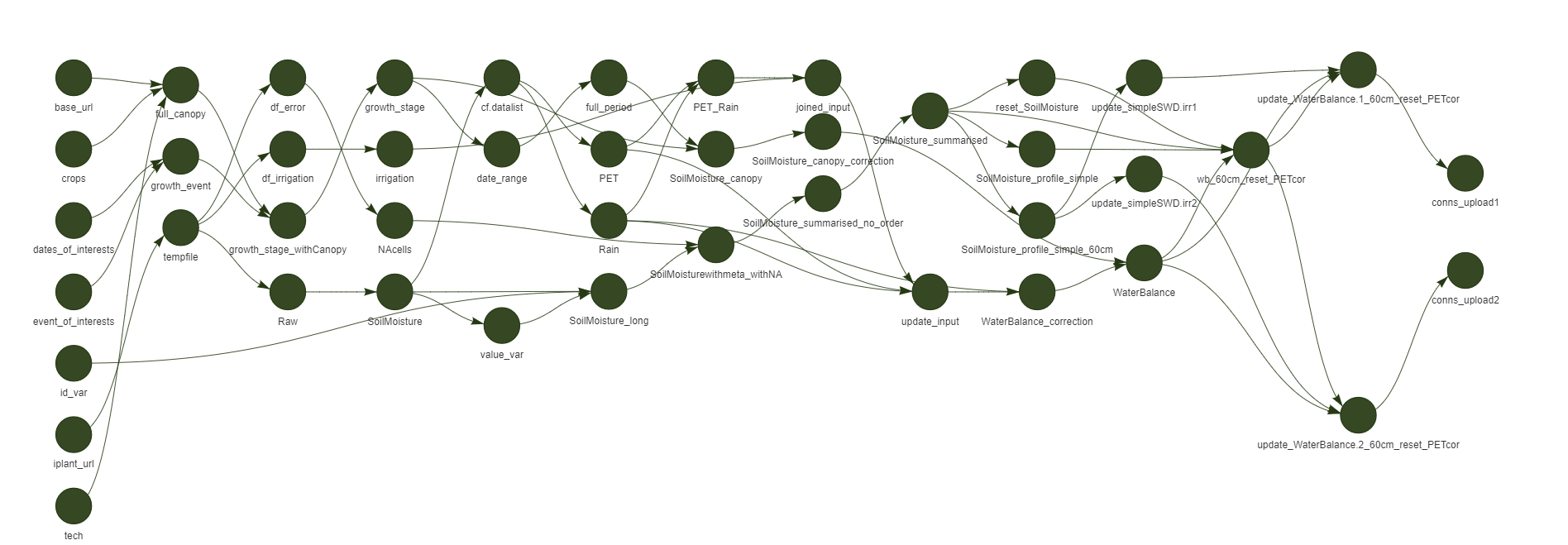


Supplementary Table 1

| Resources | Links |
| --- | --- |
| The GitHub repository | <https://github.com/frank0434/MethodXCodeRepository> |
| Interactive workflow diagram | <https://github.com/frank0434/MethodXCodeRepository/blob/methodx/webdag.html> |
| The Docker compose file | <https://github.com/frank0434/MethodXCodeRepository/blob/methodx/container/docker-compose.yml> |
| The user interface layout file | [Grafana user interface](https://github.com/frank0434/MethodXCodeRepository/blob/methodx/container/Canopy%20and%20Soil%20Water%20Balance-1644876429603.json) |

Supplementary Information 1. Steps to deploy Grafana user interface as Docker service and reproduce the demonstrated graphs

1. Installation of Docker software. Please refer to the website <https://www.docker.com/get-started/>
2. Invoke a terminal (For example, PowerShell) and navigate to the directory where the Docker-compose file is located.


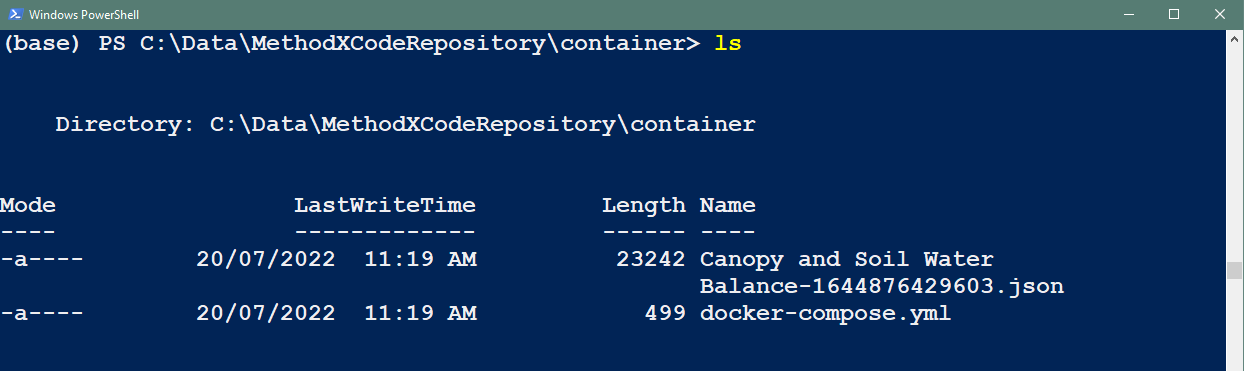


1. Use the command “**docker --version**” to verify that the docker service is properly installed and working.


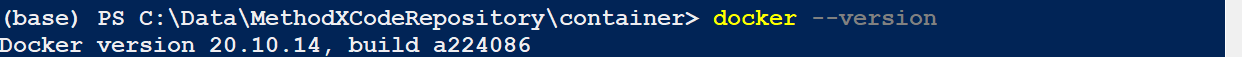


1. Run the command “docker-compose up -d” to build the container and Grafana service.


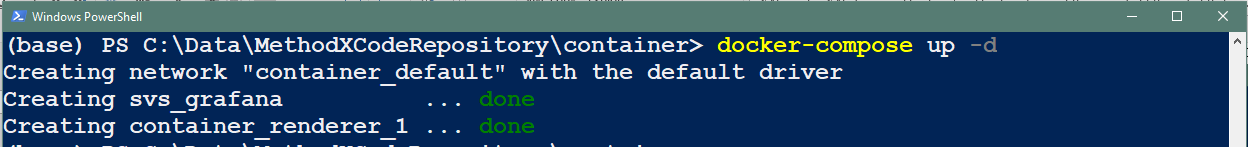


1. Access Grafana user interface via localhost (http://localhost:3000/) or server address (http://<name or IP address>:3000/).
2. Log in as admin with the password “admin” and update a secure password.
3. Add a data source under the **configuration** tab. For more details, refer to <https://grafana.com/docs/grafana/latest/datasources/add-a-data-source/>. Please note that the name of the data source has to match the name in the user interface layout file to allow the UI layout to access the data source. The current name of the data source is “MPI_SVS”. This can be changed in the JSON file as indicated in the link [here](https://github.com/frank0434/MethodXCodeRepository/blob/0043215e5665904cba0a926a4c57befb330befa2/container/Canopy%20and%20Soil%20Water%20Balance-1644876429603.json#L26).
4. Click the **Import tab** within the **Create** tab. For more details, refer to <https://grafana.com/docs/grafana/latest/dashboards/export-import/#import-dashboard>.
5. Upload the JSON file in the container folder.
6. For more about improving the visualisation and dashboard, refer to <https://grafana.com/docs/grafana/next/visualizations/>.
7. To export a static graph from one panel, click the panel header to trigger the dropdown option list and select the share tab. A dialogue window (see below) will display. The option “Direct link rendered image” will output a static png file from the panel selected.


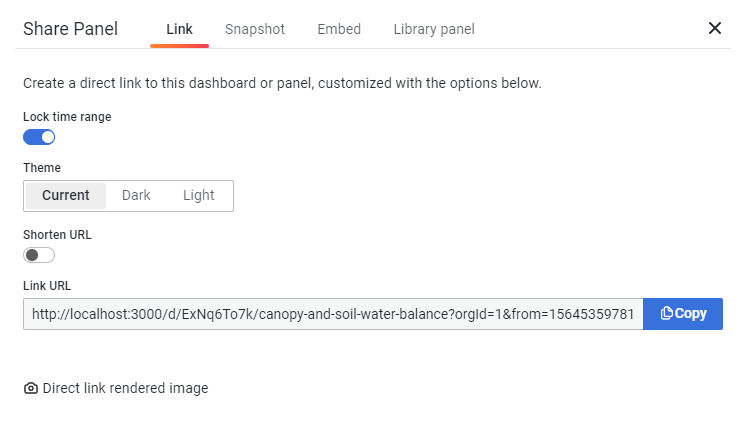

Supplement: Supplementary file 1 [file mmc1.docx]
